# Distance of bipolar re-referencing imparts nonlinear frequency-specific influences on intracranial recording signal measurements

**DOI:** 10.1088/1741-2552/ae1b3b

**Published:** 2025-11-26

**Authors:** David J Caldwell, Devon Krish, Edward F Chang, Jonathan K Kleen

**Affiliations:** 1 Department of Neurological Surgery, University of California San Francisco, San Francisco, CA 94143, United States of America; 2 Department of Neurology, University of California San Francisco, San Francisco, CA 94143, United States of America; 3 Weill Institute for Neurosciences, University of California San Francisco, San Francisco, CA 94158, United States of America

**Keywords:** electroencephalography, stereo-EEG, depth, grid, epilepsy, sampling

## Abstract

*Obective.* Bipolar re-referencing (BPRR), in which one electrode’s signal is subtracted from a neighboring electrode to produce a differential signal, can improve signal readability and refine localization for intracranial electroencephalography. There is wide variation in manufactured electrode array spacing, yet how BPRR affects specific frequencies at precise inter-electrode distances has not been systematically evaluated. *Approach.* Intracranial recordings with uniquely large numbers of electrodes were obtained for sixteen patients with drug-resistant epilepsy. We evaluated combinations of high-density subdural grid, depth, and strip electrodes (*n* = 3,664, 742, and 336) with manufactured linear inter-electrode distances of 4, 5, and 10 mm, respectively. BPRR was performed using all possible electrode pairs (*n* = 445 305 grid, 16 004 depth, 3278 strip) spanning distances from 2–60 mm. Multi-taper power spectra were generated separately for grid, depth, and strip contacts. Distances were consolidated across patients and anatomical areas for generalizability, and distance-related influences on task-related brain activity and quantitative interictal epileptiform discharge localization were evaluated. *Main results.* We identified 8 mm as a consistent reversal point for BPRR, below which low-frequency signals (<30 Hz) had consistently decreased power, and higher frequencies had increased power. Larger distances increased all broadband (2–200 Hz) signals. Task-related increases in superior temporal gyrus 50–200 Hz activity were consistently enhanced across 4–40 mm bipolar distances. There were non-significant difference trends between 4 and 8 mm re-referencing on epileptiform discharge detection. *Significance.* BPRR distance imposed specific transition points for distance and frequency (roughly 8 mm and ∼30 Hz, respectively) that produced differential effects on measurements of signal power. The consistency across brain regions and electrode types (depth, subdural) suggests these influences are physical brain bio-signal properties, potentially related to spatial wavelength of periodic oscillations in lower frequencies in contrast to more aperiodic activity in higher frequencies. A distance-frequency relation map is provided to help optimize neural signal biomarker quality for intracranial applications by guiding strategic re-referencing distance selection.

## 1. Introduction

Voltage signals from intracranial EEG (ICEEG) electrodes are recorded on differential amplifiers in reference to a single electrode (referential recordings). Following digitization, re-referencing is frequently used for clinical or research purposes. Most commonly, this involves bipolar re-referencing (BPRR), a spatial derivative in which the recorded trace from an electrode is subtracted from the trace of its consecutive neighboring electrode (i.e. bipolar pair) to yield a single new trace.

BPRR profoundly changes the properties of the resulting trace [1]. Key motivations for its use are the reduction or removal of artifact shared between the electrodes and volume-conducted neural activity from distant sources [2–4]. These direct effects are generally intended to improve the detection of locally-generated activity for localization, including focal epileptiform waveforms which often have high-frequency and/or sharp components [5–7]. BPRR also holds key roles in research applications, including as a spatial filter for locally-generated activity. Specifically, low frequencies are thought to have a larger electrical field in space and are thus largely subtracted out between the electrode pairs. Higher frequencies (e.g. high gamma activity, a surrogate for local neuronal population activity) on the other hand likely represent a mixture of oscillatory (‘periodic’) and non-oscillatory (‘aperiodic’) background activity picked up across many frequencies, potentially more likely to differ from electrode to electrode and thus better distilled with BPRR (higher signal-to-noise, or SNR) [8–10]. Hence, bipolar montages improve finer-scale localization of normal and abnormal neural activity by influencing signal measurement, supporting their frequent use in research and clinical contexts.

Intracranial recording electrodes rely on manufacturer-determined inter-electrode distances, typically 5 mm for depth electrodes and 8 or 10 mm for subdural grid and strip electrodes [11]. However, it is unknown how the precise physical distances in 3D space (Euclidean) systematically affect frequency-based biomarkers and the localization of epileptiform activity. This is despite the importance of anatomic seizure-onset zone margins which are influenced clinically by electrode density [12], not to mention detection of focal epileptiform activity important for epilepsy surgery decision-making [13, 14] including high-frequency oscillations and interictal epileptiform discharges (IEDs) which also have important clinical relevance for successful epilepsy surgery [14–16]. BPRR is also applied in emerging chronic intracranial ambulatory monitoring applications for neurological conditions (epilepsy, movement disorders, and others) to improve local signal-to-noise ratio. Yet again, the precise distances of manufactured inter-electrode distances are technically arbitrary, and bipolar electrode pairs are often selected by trial-and-error and visual inspection to obtain sufficient signals [1, 17–19].

Quantitative evaluation of various BPRR Euclidean distances would guide optimization of spectral biomarker detection for closed loop systems [20–24], analysis of parameter trends over time [9, 10, 25, 26], and to improve clinical ICEEG interpretation [3]. While previous studies have assessed correlations of power and other features between adjacent and remote electrodes [27, 28], and the impact of re-referencing on functional connectivity,^3^ here we instead systematically vary bipolar pair distance ***prior to*** power spectral analysis and examine distance-related influences across the broadband spectrum. We also evaluate whether different distances influenced the spatial extent and temporal durations of IED detection for additional clinical relevance. Since variation across patients and anatomical sites is likely, we leveraged a dataset of research-grade interictal ICEEG recordings with massive channel counts. This included standard and high-density inter-electrode spacing, across superficial and deep sites stratified by array type, to understand the general influences of BPRR distance on ICEEG signals.

## 2. Methods

### 2.1. Study population and recordings

**Figure 1.**
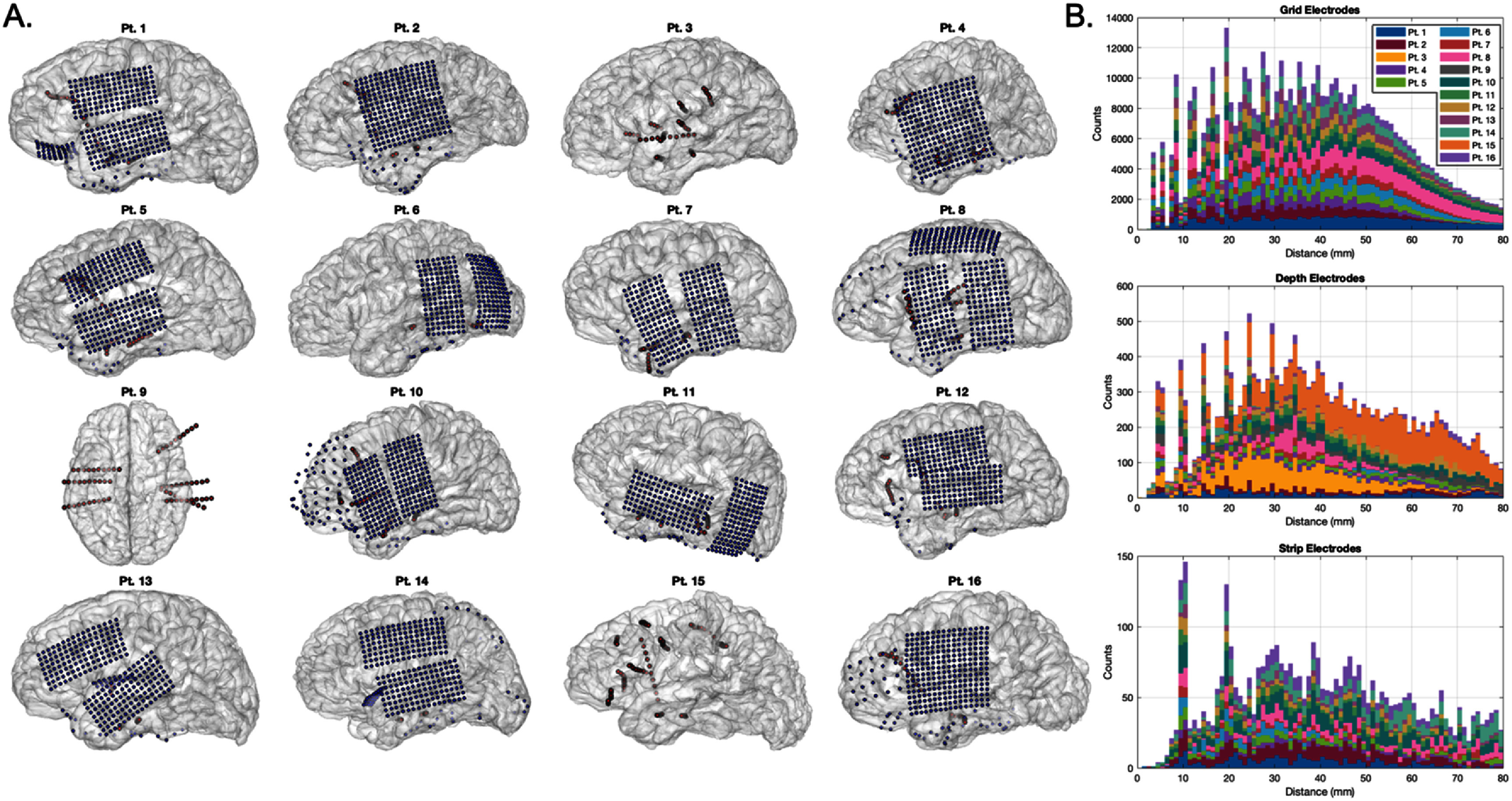
Electrode coverages and distributions of bipolar pair distance. (A) Cortical reconstructions based on pre-implantation MRI scans for sixteen subjects included in the main spectral analyses herein. Implanted electrodes co-registered using post-implantation head CT scans, with blue dots representing high-density grids (4 mm inter-electrode spacing) and strips (10 mm spacing), and red dots representing penetrating depth electrodes (5 mm spacing). (B) Histograms showing the Euclidean distance-based distributions of all possible bipolar pairs for grid (top), depth (middle), and strip (bottom) electrode pairs, stratified by patients (colors). Peaks in shorter distances represent typical manufactured spacing of each component type (grids: 4 mm, depths: 5 mm, strips: 10 mm) and multiples of this base spacing, with variability due to exact positioning in 3D space (e.g. bending of electrode arrays around or within brain anatomy).

We utilized intracranial recordings from sixteen patients performed for drug-resistant epilepsy at UCSF (seven women, nine men; table 1). All patients had depth electrodes (5 mm inter-electrode distance). Nine also had combinations of simultaneous subdural strips (10 mm spacing) and high-density grid arrays (4 mm spacing), recently described as ‘hybrid recordings’ [29]. Electrode coverage for the patients included in the main spectral analyses are shown in figure 1(A). Depth electrodes (10-contact) and strip electrodes (4 or 6-contact) were manufactured by Ad-tech Medical Instrument Corporation (Oak Creek, WI) and grid electrodes were manufactured by PMT Corporation (Chanhassen, MN). The research was conducted in accordance with the principles embodied in the Declaration of Helsinki and in accordance with local statutory requirements. All participants (all were over 18 years old) gave written informed consent to participate in the study. This work was approved by the UCSF Institutional Review Board (protocol #10-03842).

**Table 1.** Participants and ICEEG windows used for analysis. Participants with less than 50 IEDs were not included in the IED analysis (N/A in last column).

Participant	Sex	Number of electrodes	Number of baseline windows	Number of IED windows
1	Male	320	876	90
2	Male	328	582	N/A
3	Female	78	1836	N/A
4	Female	314	2067	N/A
5	Female	318	669	395
6	Male	314	1164	N/A
7	Female	302	319	N/A
8	Female	466	1295	N/A
9	Female	70	1264	153
10	Male	384	1104	233
11	Female	320	1565	194
12	Male	328	391	578
13	Male	330	1495	103
14	Male	362	1569	412
15	Male	110	1814	204
16	Male	350	992	147

Neural data was recorded in 2–10 sessions per patient, with sessions ranging from 0.5 min to 8.8 min in duration. We recorded from all available intracranial electrodes using a multichannel amplifier optically connected to a digital amplifier and analog-digital converter with up to 512-channel capacity manufactured by Tucker-Davis Technologies (Alachua, FL). An electrode contact from a 4-contact subgaleal strip was used as the reference for all channels, which were digitized at a sampling rate of 3052 Hz. Channels with poor signal quality, noise, or substantial artifacts in the local field potentials (LFPs) upon manual review were excluded from further analyses. ICEEG data was screened for artifacts (electrical, muscle, movement; manually identified by J.K.K.) and their onsets through offsets were annotated. The onsets and offsets of IEDs were also annotated. This IED detection process first leveraged an automated line length detection method [30] followed by manual screening by an epileptologist (J.K.K.) identifying epileptiform sharp waves, and transient bursts with epileptiform features (sharp and/or spike morphology with or without overlying high frequency activity, including repetitive bursts). Each IED was aligned to the data point with the maximum absolute slope across channels (absolute value of the ECoG derivative) as the center of 1 s ‘IED windows’ including 0.5 s before and after.

Each recording was parsed into 1 s consecutive non-overlapping windows (baseline windows). Any windows overlapping with the duration (onset through offset) of manually annotated IEDs were omitted from all analyses (except the IED-focused analysis in which the 1 s windows were re-centered around each, as described above). Windows overlapping with the durations of speech stimuli (onset through offset) were labeled for the task related analyses. An anti-aliasing filter (<255 Hz low-pass Butterworth filter) was applied, followed by down-sampling to 512 Hz, and subsequently notch filtering to at 60 Hz and its harmonics (120, 180, 240 Hz) to address line noise.

### 2.2. BPRR

**Figure 2.**
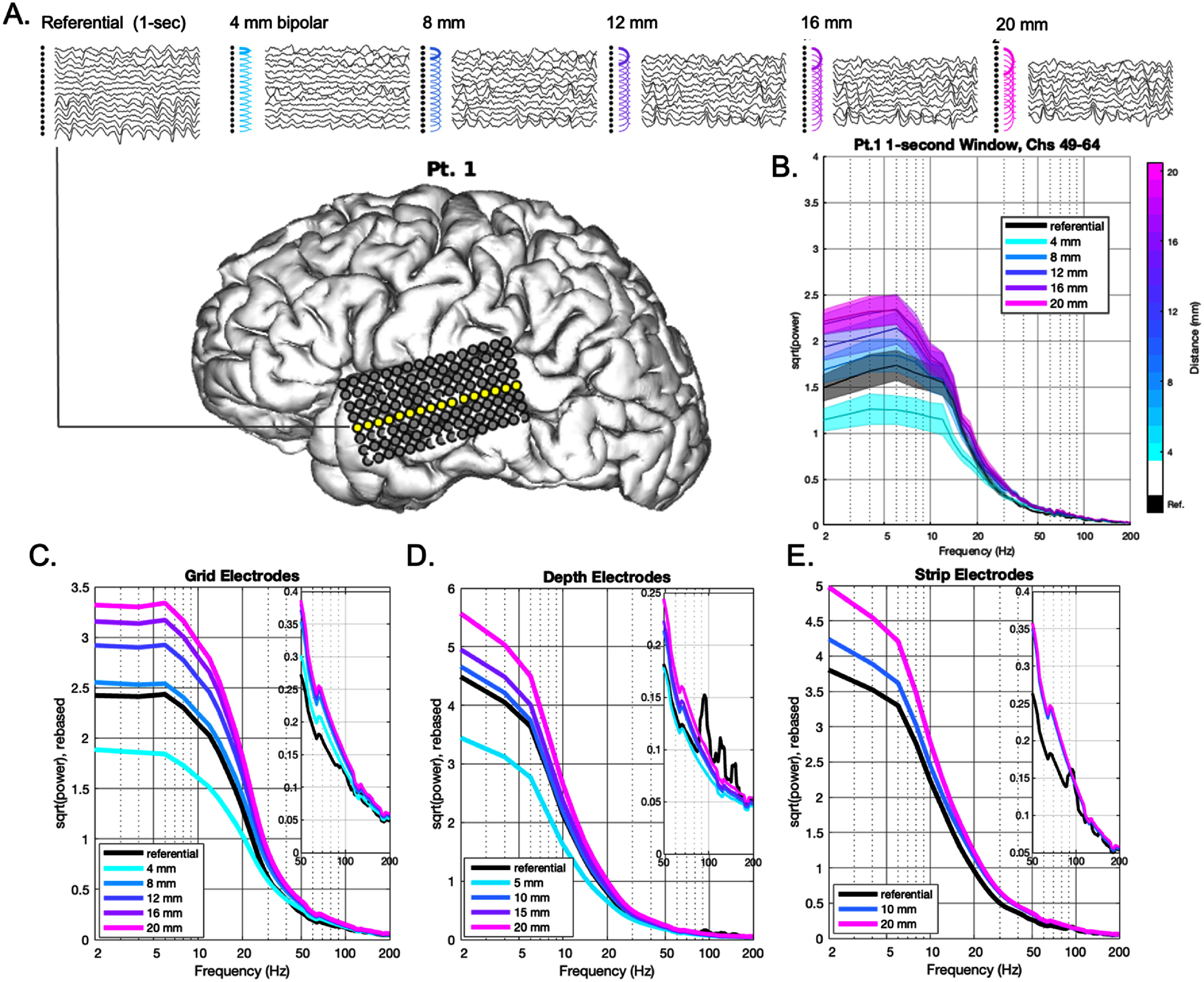
Linear bipolar re-referencing according to distance. (A) Time series traces for a row of individual channels with increasing bipolar distances for a single subject. (B) Power spectrum corresponding to a 1 s window generated by the channels in (A), highlighting the reduction in power in lower frequencies for the 4 mm bipolar re-referencing distance, and increases of higher frequency activity. For greater BPRR distances, power was increased for all frequencies. Solid lines represent the mean signal, with surrounding shading representing the standard error of the mean. (C) Mean power spectra (variance omitted for visualization purposes; individual patients shown in supplementary figure 1) from 2 to 200 Hz derived from grid electrodes, averaged across all patients and rebased by subtracting the median of the referential signal, averaged across all electrodes within a given subject, between 2 and 10 Hz. We define this as the “rebased’ signal. Surrounding shading represents standard error of the mean across subjects. (D) and (E) are similar to (C) but derived from depth and strip electrodes respectively. High frequency artifact (peaks in referential spectra >75 Hz) in the original referential recordings can be noted particularly in depth and strip electrodes, which was inherent across electrodes and which is therefore naturally removed in BPRR spectra.

Raw referential voltages were systematically re-referenced in bipolar style at various distances for each participant. Specifically, for a given electrode pair, we subtracted one electrode’s referential signal from the other electrode’s referential signal prior to spectral analysis and documented the distance. We evaluated pairs within the same components (e.g. depth electrode and another depth electrodes), but not between components (e.g. grid electrode and a depth electrode) to avoid confounds including different contact sizes, materials, and manufacturers. Spectral data from bipolar pairs for each distance condition were binned and averaged at regular distances to enable neural activity sampling from diverse anatomical regions (gyri, sulci, fissures, lobes, deep and superficial sites), improving generalizability of any regularities detected in the results. By design no distinction was made between contacts in white and gray matter. We used two BPRR strategies in the analysis:

• ***Linear ordinal BPRR***: as in typical clinical practice, linear ordinal BPRR was performed at different distances by skipping one to four electrodes between bipolar pairs (figure 2(A)) along the linear orientation for the different components: depth probes, strips, and for each row of grids. The recorded distance was expressed as the ordinal number of inter-electrode distances skipped for a bipolar pair multiplied by the manufactured inter-electrode spacing (i.e. non-Euclidean).
• ***Omni-directional BPRR***: a more spatially detailed evaluation of physical distance in 3D space was performed using an omni-directional re-referencing procedure, in which each electrode was paired with every other possible electrode prior to spectral analysis. The Euclidean distance was recorded for each pair based on digital reconstruction using post-implantation head CT imaging.

### 2.3. Spectral analysis

Any baseline windows overlapping with these IED-centered windows, and segments containing external noise, muscle activity, or other artifact, were excluded from spectral analysis results due to risks of spectral contamination [5]. We calculated multi-taper power spectra using the Chronux toolbox [31, 32] for frequencies from 2–200 Hz in 1 Hz intervals from non-overlapping 1 s baseline ICEEG windows across all recordings. When referring to canonical frequency bands, we refer to their frequency ranges in this study as delta: 2–4 Hz, theta: 4–8 Hz, alpha: 8–13 Hz, beta: 13–25 Hz, gamma: 25–50 Hz, and high gamma: 50–200 Hz.

### 2.4. IED analysis

An additional quantitative analysis using linear ordinal BPRR was applied to IED localization, given direct relevance to clinical practice. For each grid row, every other electrode (‘base electrodes’) in the high-density underwent BPRR with either its first adjacent (4 mm away) or its second adjacent (8 mm away) electrode. This enabled both high- and low-density sampling conditions [12] while using the same base electrodes for the analysis (figure 6). The high-density and low-density linear ordinal BPRR schemes were applied to the IED analysis by performing an instantaneous line length transform (absolute value of the signal derivative) on both the IED-centered windows of data described above and a larger number of baseline windows without IEDs (table 1) [30, 33]. To ensure sufficient statistical power only participants with >50 detected IEDs were included; see table 1). IED-aligned windows were evaluated against baseline windows using two sided, channel-wise cluster-based permutation tests [34] to determine the temporal and spatial extents of the IEDs, quantified as the total duration (timepoints) of the IEDs rising above baseline and the number of bipolar channels involved in IEDs.

### 2.5. Speech analysis

For the two patients with 16 × 16 grid electrode coverage spanning the superior temporal gyrus (STG), data windows when patients were listening to auditory stimuli were extracted, log transformed, and subsequently z-scored by frequency across binned distance. Channel-wise, two-sided cluster based permutation tests were used to compare spectral composition features for the auditory naming speech stimulus task related data compared to baseline. Analysis was restricted to electrodes in the STG given the known role of the middle- and posterior-STG in speech processing (distances up to 40 mm tested in line with typical functional extent of STG for speech processing) [35, 36].

### 2.6. Statistical analysis

Spectra calculated on omnidirectional BPRR were square root-transformed, and then z-scored by frequency across binned bipolar distances since the primary goal of the study was to understand distance-related influences. For the IED analyses within each subject, we then corrected across all channels with significant IEDs as assessed through the permutation test for a false discovery rate of *p* < 0.05 using the Benjamini–Hochberg approach [37]. Spatial and temporal IED analyses were statistically compared between high-density and low-density conditions using non-parametric signed-rank tests (Wilcoxon). The comparisons across different line length algorithm conditions were additionally corrected with a Bonferroni correction. Data processing and all statistical analyses were performed in MATLAB version 2024b (The MathWorks, Inc.; Natick, MA), except for the Wilcoxon signed-rank tests which were performed in Python (version 3.9.12).

## 3. Results

There were 3664 grid electrodes, 742 depth electrodes, and 336 strip intracranial electrodes across the participant cohort evaluated in the study (figure 1(A)). Following removal of bad channels (due to artifact; see methods), re-referencing using all possible within-component electrodes spanning distances from 2 to 60 mm revealed 445 305 grid electrode pairs, 16 004 depth electrode pairs, and 3278 strip electrode pairs (figure 1(B)). The placement and orientation of electrodes varied across patients in anatomic location and orientation, including the spanning of sulci and fissures, as well as white matter and intralaminar cortex (depth electrodes) versus cortical surface (grids and strips) as illustrated in figure 2(A).

### 3.1. Linear ordinal BPRR distance

The relationship between frequency and power as a function of linear ordinal BPRR distance is illustrated in figure 2, including an example of a single 1 s time window for one patient in figures 2(A) and (B). All bipolar pairs along parallel grid rows were assessed independently (i.e. electrodes #1–16, #17–32, and so on). For each linear ordinal bipolar distance (and referential) power spectrum, we averaged across time windows (within-patient; number of windows per patient shown in table 1), and then averaged across patients and illustrated as colored lines representing the mean signal in figures 2(C)–(E). This mean signal was the spectra from 2 to 200 Hz derived from either grid, depth, or strip electrodes, averaged across all patients. To enabling comparison while accounting for patient-specific differences, power spectra curves were adjusted by subtracting the median of the referential signal, averaged across all electrodes within a given subject between 2–10 Hz. This rebased signal enabled relative comparisons spectral features across subjects, electrodes, and frequencies. The calculated power levels of low frequencies between roughly 2–30 Hz (i.e. delta, theta, alpha, beta) were attenuated at a 4 mm distance, but at an 8 mm distance this reverted, similar to original referential levels (figure 2(C)). These same frequencies were increased in power at larger BPRR distances of 12–20 mm. In contrast, frequencies above approximately 30 Hz (gamma, high gamma) were increased at all evaluated ordinal distances.

We hypothesized that distance-dependent influences on frequency power would be similar regardless of whether evaluated on grid, depth, or strip electrode pairs. Therefore, we next used single row electrode pairs from depth and strip components to similarly assess power as a function of linear ordinal BPRR distances. At a very short distance of 5 mm (depth electrode pairs only), an attenuation of power for frequencies less than approximately 30 Hz was again observed (similar to 4 mm using grid electrodes; shorter distances were not assessable on strips due to a manufactured inter-electrode distance of 10 mm). Virtually all frequencies were increased at longer linear distances of 10 mm and above for both depth (figure 2(D)) and strip (figure 2(E)) electrode pairs. This was again similar to grid electrode pairs, suggesting this to be a physical property of power-frequency dynamics as a function of BPRR distance.

Default ranges of canonical frequency bands (e.g. delta, theta, etc. as listed in methods) are used ubiquitously in ICEEG neurophysiology, including as biomarkers in closed loop systems [20, 21, 23, 24]. Therefore, we next delimited frequencies into these bands as a general, practical evaluation of distance-dependent influences. We adjusted across patients by subtracting (rebasing) the average referential power across grid electrodes within each patient from all BPRR distances. The results were consistent with the continuous frequency measures in figure 2. Namely, delta power was decreased at 4 mm distances, near-similar to referential at 8 mm, and increased at distances of 12 mm and higher (*p* < 0.001, FDR corrected, Wilcoxin signed rank tests between mean powers averaged across electrodes within each subject, within each frequency band, and the referential signal; supplementary table 1, Supplementary figure 2). These patterns were similar for other ‘low frequency’ ranges (theta, alpha, and beta), including beta power increasing at 8 mm distance (*p* < 0.001, Wilcoxin signed rank tests). In contrast, higher frequencies (gamma and high gamma) were increased at progressively larger distances at 8 mm and longer (*p* < 0.001, Wilcoxin signed rank tests). The specific numeric results (median, inter-quartile range, effect size, FDR-corrected *p*-value) for each band at each distance are located in supplementary table 1.

### 3.2. Omnidirectional bipolar pair distance on high density grids

**Figure 3.**
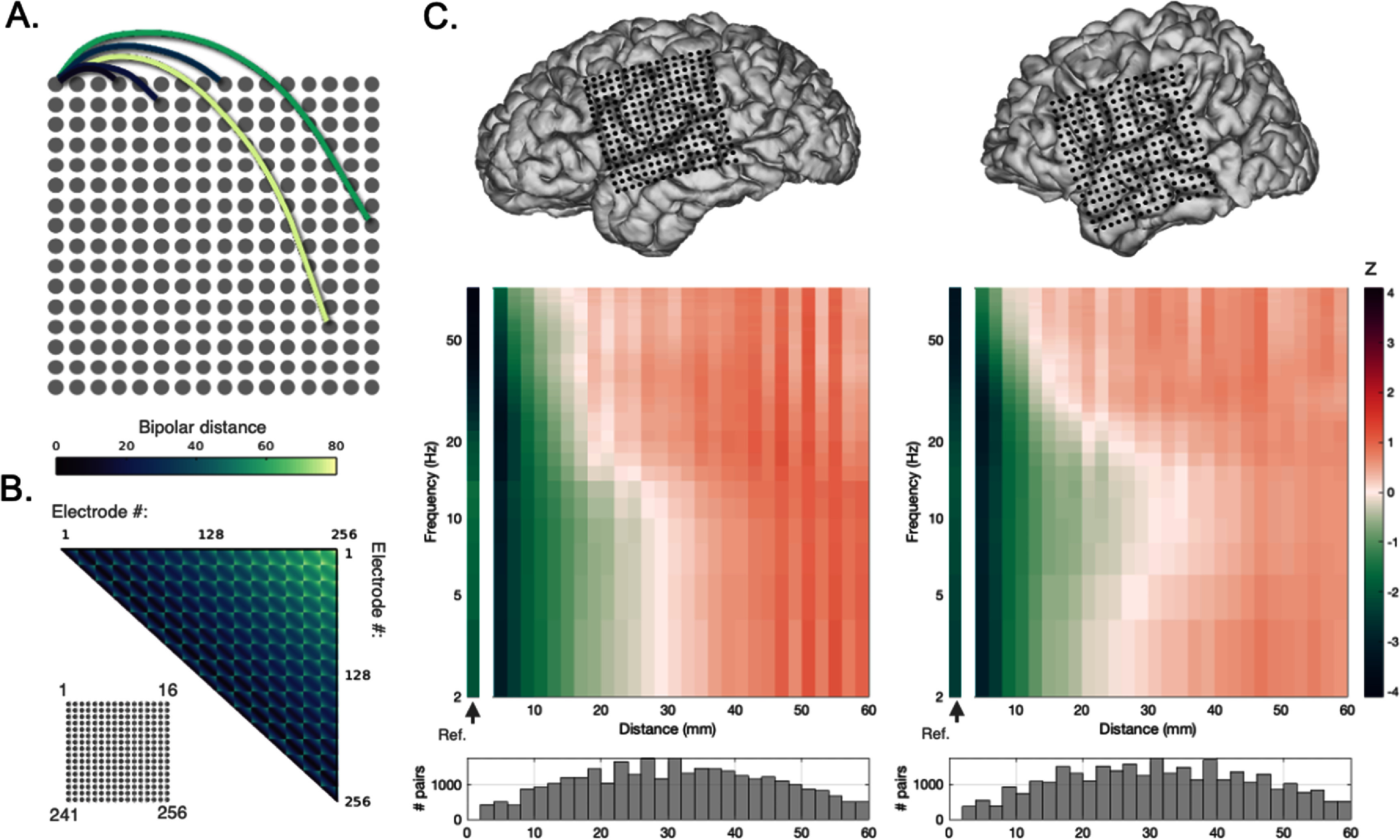
Omnidirectional bipolar re-referencing. (A) Approach for omnidirectional BPRR is illustrated with five example bipolar pairs indicated by lines with colors denoting their inter-electrode distance. (B) Euclidean distance between all possible bipolar pairs of a 16 × 16 channel grid (4 mm pitch) over the cortical surface of an example patient (Pt. 2), calculated using their 3D (x–y–z) coordinates. Color scheme as in (A), (C) spectral power-distance maps for two subjects with uninterrupted 16 × 16 high density ICEEG arrays (Pt. 2 at left, Pt. 4 at right; electrode locations on respective brain reconstructions shown at top). Relative spectral power (square-root transformed then z-scored by frequency) averaged across all time windows is illustrated as a function of bipolar pair Euclidean distance (2 mm bins). The non-linear effects of distance and frequency on power are evident as a crescent-shaped effect (decreased power for 2–30 Hz at shorter distances but neutralizes and then increase at higher distances, with nadir at ∼10 Hz with its distance transition point the longest at ∼30 mm). This is observed as a similar distances-by-frequency pattern in both participants.

While linear ordinal BPRR on consecutive contacts (i.e. discretized sampling of bipolar distances) is commonly used in clinical practice and many research applications, using 3D Euclidean distance (figure 3(A)) provides more accurate physical space modeling for detailed understanding of BPRR distance influences on power. It also enables the inclusion of far more electrode pairs (e.g. across grid rows and columns, or between depths, or between strips). We first iterated across all possible electrode pairs on 16 × 16 high density grids, producing a large number of possible bipolar pair distances from 4 to 85 mm (figure 3(B); note: since distances above 60 mm were relatively rare, we omitted these pairs to avoid noise in the analysis). Bipolar distances in this analysis were calculated from the three-dimensional reconstructed electrode locations co-registered in the post-implantation reconstructions. This accounts for physical variation such as minor or major curvatures of the electrode arrays in the brain and on its surface, and BPRR between arrays. Similar to the linear distance analysis above, by resampling the same electrodes with numerous orientations and modeling across a large number of distances, we could minimize the influences of anatomical features, including gyri, sulci, and fissures on the spectral analysis. We averaged square root power across bipolar distance pairs in 2 mm bins and plotted the means by frequency (figure 3(C)).

Nearly all frequencies showed increased power at distances above 10–14 mm, as illustrated in figure 3(C) (similar to the linear distance analyses in figure 2 and supplementary figure 1). However, the effect of distance on power according to frequency was nonlinear. Specifically, there was a ‘crescent-shaped’ distribution of mean power: low frequencies roughly 5–30 Hz (theta, alpha, beta) increased more gradually as a function of distances between 20–45 mm distances. Meanwhile, higher frequencies above 30 Hz were relatively increased at these distances. Notably, at distances less than 10 mm, we observed a relative attenuation in power for frequencies between 2–30 Hz, consistent in physical space with our linear analysis in figure 2 (and supplementary figure 1). The nonlinear crescent-shaped relation of power as a function of distance and frequency was consistent across both patients with 16 × 16 grids, despite only moderate overlap in the anatomical regions covered.

### 3.3. Relative change from referential signals

**Figure 4.**
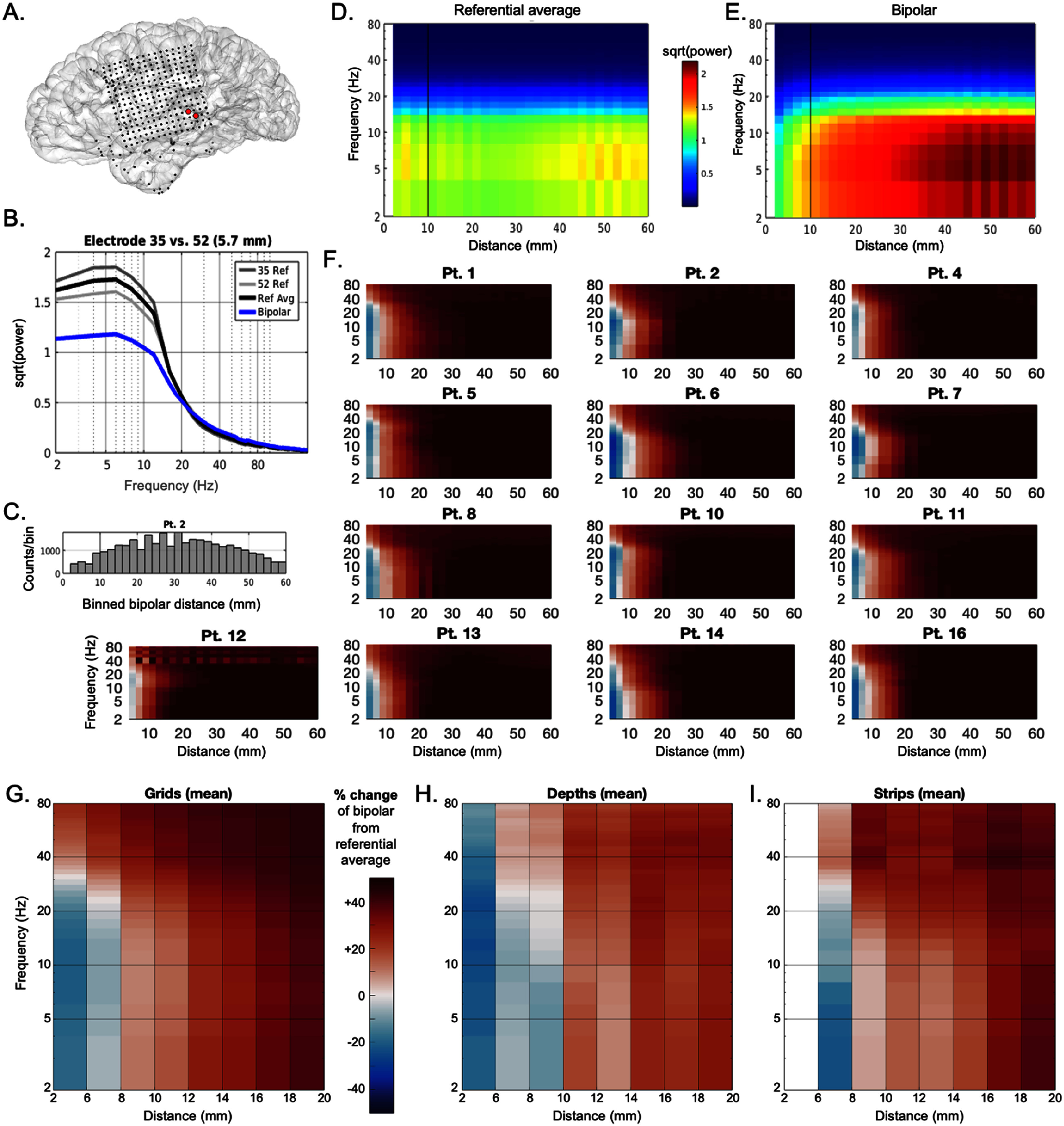
Relative changes in power as a function of BPRR distance. (A) Two example adjacent channels from Pt. 2. (B) Power spectra averaged across all time windows for the example referential channels in (A) (gray lines), their average (black line), and their bipolar pair signal (blue line). (C) Distribution of all possible bipolar grid electrode pairs for the patient in (A), (D). Power spectra averages for grid electrode pairs (square root of power, average of both referential signals, as with black line in B) across all 1 s windows for the patient in (A)–(C), binned by inter-electrode distance (2 mm bins averages). (E) Similar to (D) yet using BPRR signal (as with blue line in (B) for all pairs. (F) Percent changes in power (magnitude of increase or decrease) at each frequency and each distance for individual patients (panels), calculated using mean BPRR spectrum of contributing grid electrode pairs normalized by their referential pair average spectra (i.e. for each patient, (D) minus (E), then divided by (E). Relative changes from referential power for individual patients using depth and strip electrodes is displayed in supplementary figure 3. (G) Global distance-frequency relation map (averaged across all patients in F) of relative change of spectral power in BPRR vs referential pair average signals for all grid electrode pairs. H. Similar to G, but for depth electrode pairs only. I. Similar to (G), but for strip electrode pairs only (white sections lack sufficient pairs for conclusive results, refer to figure 1(B) bottom panel).

We then sought to understand the degree to which spectral power from two electrodes in referential recordings is directly affected as a function of BPRR distance. To accomplish this, for each pair of electrodes at a given distance, we subtracted the bipolar signal’s power spectrum from the mean of the referential signal spectra from the contributing electrodes (figure 4(B)). We first used this approach on grid electrodes, similar to the analysis in figure 3, finding a similar crescent-shaped pattern of attenuation and augmentation (figures 4(F) and (G)). Specifically,

• Low frequencies between 2–30 Hz (delta, theta, alpha, beta) showed power attenuations as low as approximately 20% at distances less than 8 mm, though power became roughly equivalent to original referential signals at distances between 8–10 mm and became increased up to 58% at longer distances of 52–54 mm.
• Higher frequencies between 30–200 Hz showed power increases of 5.5%–30% below 8 mm and stronger increases at longer distances, plateauing at approximately 56 mm (46% increase).
• We performed this same analysis on strip and depth electrodes, corroborating similar effects of bipolar distance on frequency-specific power levels (figures 4(H)–(I), supplementary figure 3).

### 3.4. Differences in task-related spectral power

**Figure 5.**
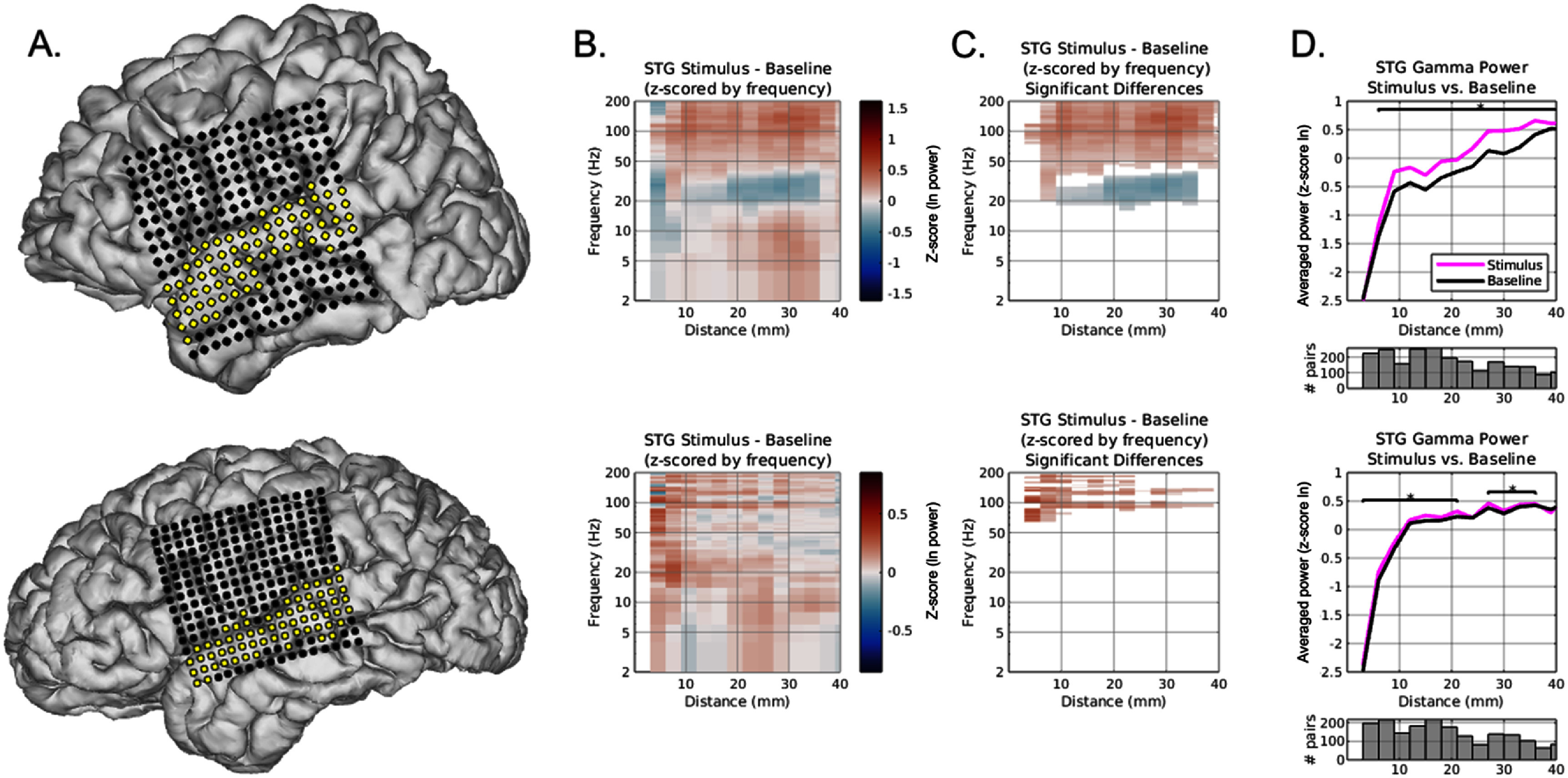
Influences of bipolar distance on neural population activity in STG during an auditory naming speech stimulus. (A) Electrode grid array on individual brain reconstructions, with electrodes over the STG shown in yellow for the two patients with a full continuous 16 × 16 array (top, Pt. 2; bottom, Pt. 4). (B) For each frequency and each bipolar distance, the difference in neural activity between stimulus minus baseline is shown (specifically, stimulus baseline inter-trial periods subtracted from periods in which the patient was hearing an acoustic stimulus [auditory naming sentence]). The result here is averaged across all electrode pairs in each distance bin. (C) Similar to (B) yet showing only data for which a cluster-based permutation test between the conditions (stimulus vs. baseline periods) was statistically significant, showing that the ability to discern a change (increase) in high gamma (50–200 Hz) neural activity in the STG (compared to baseline with no stimulus) is enhanced at bipolar distances particularly between roughly 4–40 mm. There was a significant decrease in 20–40 Hz power in one of the patients only at distances below 40 mm. D. Using the average across 50–200 Hz as a classic ‘high gamma’ metric demonstrates the effects shown in the more detailed B and C plots, underscoring that the frequencies chosen to assess local neuronal population activity have some sensitivity to bipolar re-referencing distance.

Relations between the signals recorded at electrodes, and thus between the neural populations they sample, can depend on the network state, such as whether the participant is involved in a behavioral task.^16^ We focused on all electrodes located in the STG region, an area involved in receptive speech processing, and evaluated for any differences in bipolar power between when participants were actively listening to spoken sentences (Adapted Auditory Naming acoustic sentence stimuli) [38] or at rest (figures 5(a) and (B)). Replicating prior work [35], we found increased 50–200 Hz activity while patients were listening to stimuli relative to rest periods (figure 5(C)). This effect increased at higher bipolar pair distances (10–40 mm; *p* < 0.05, cluster-based permutation test; figure 5(D)) and it was less pronounced at the smallest available BPRR distances (figure 5(D)) and depended upon the specific frequency with variability between the two patients (figure 5(C)). We also noted a decrement in beta frequencies at 10–35 mm distances while listening to stimuli compared to rest in one of the two participants (figure 5(C), lower panel).

### 3.5. IED detection using high- vs low-density recordings

**Figure 6.**
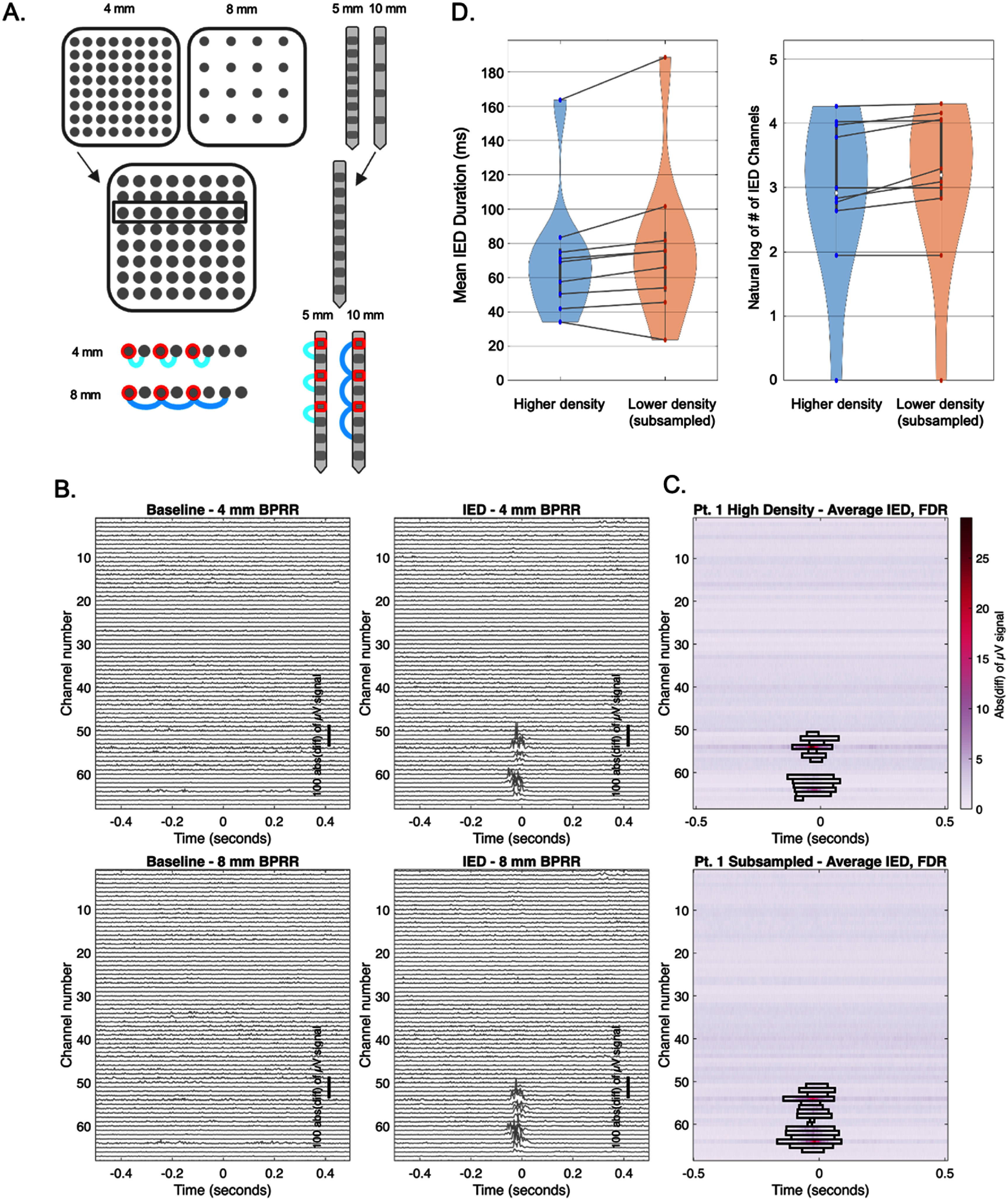
Detection of IEDs using higher-density vs. lower density sampling. (A) BPRR was performed using the original 4 mm spacing (‘high-density’) for pairs (left grid) or by skipping every other electrode to artificially approximate 8 mm spacing (‘low-density’) for pairs (right grid). The same procedure was performed for depth electrodes (5 and 10 mm spacing, respectively). (B) Instantaneous line length transform of 4 mm and 8 mm bipolar channels (top and bottom respectively), illustrating 1 s window examples of baseline activity (left) and a IED (right, IED centered at middle of panel using maximal absolute slope) for Pt. 1(C) Average Instantaneous line length transform, with outlines showing spatial (electrode) and temporal (timepoint sections) data points surpassing significance relative to randomly drawn baseline windows (*p* < 0.05, cluster-based permutation test).(D) Violin plots illustrate no significant differences in temporal or channel-wise IED detection using the absolute derivative (instantaneous line length) across these BPRR domains after Bonferroni correction.

BPRR is often used during clinical ICEEG review for the detection of IEDs, and so here we examined whether bipolar referencing distance influenced the localization (improves the spatial signal-to-noise ratio) of IEDs. Both the duration and channel involvement (spatial extent) of IEDs are clinically relevant for annotating epileptogenic tissue, hence both were evaluated statistically. The number of IED-centered windows for analysis ranged from 90–578 per patient whereas the number of baseline windows ranged from 391–1814 (table 1). We re-referenced electrode pairs on linear grid rows, strips, and depth probes using 4 vs 8 mm (subsampled) spacing conditions, or 5 vs 10 mm for depth probes, corresponding the ‘high-density’ and ‘low-density’ conditions respectively [12] (figures 2 and 6(A)). We then used only those electrodes present in both conditions for analysis (figure 6(A)). An automated detector threshold on the line length transform of the ICEEG [30, 33], evaluating varying sliding window lengths of 100 ms, 40 ms, 20 ms, and instantaneous line length (i.e. absolute value of the first derivative of the EEG, approximating a ∼2 ms window). We found trends in IED detection (duration as assessed by mean width, *p* = 0.074 and number of channels involved, *p* = 0.016) between high- versus low-density BPRR, as shown in figures 6(B)–(D), when using the absolute derivative of the signal for illustrative purposes and Bonferroni correction accounting for testing across the four line lengths (significance specified as *p* < 0.0125). Line length window duration did not consistently affect the number of channels nor duration of IEDs detected across the high-density and subsampled schemes (supplementary table 2; supplementary figure 4).

## 4. Discussion

We systematically varied BPRR distance *prior to* calculating signal metrics, in contrast to prior literature which has compared BPRR to other re-referencing schemes, or evaluated distance-related correlations of power levels and connectivity (i.e. *after* re-referencing) [3, 4, 27, 28, 39]. To increase generalizability and account for signal variance simply from sampling different neuronal populations (i.e. different electrode sites), we leveraged massive numbers of electrodes site pairs across sixteen participants and independently tested across different recording hardware components (grids, depths, strips). This data-driven approach revealed consistent properties at specific frequencies tightly tied to physical (Euclidean) distance. These consistencies may help optimize BPRR distance selection for frequency-dependent biomarkers; for example, for a 30 Hz biomarker of interest, one might select choosing a bipolar pair spanning 8 mm distance in the region of interest to ensure this signal is accentuated and not attenuated in general (figure 4(F)).

We found that the well-known high pass filtering effect of BPRR (i.e. attenuation of low frequencies such as delta, theta, etc.) only pertained to frequencies up to roughly 30 Hz, and crucially, this was only observed at BPRR distances less than 8 mm. These effects were consistent across both linear ordinal and omnidirectional analyses (figures 2–4) and across grid, depth, and strip recording components (figures 4(F)–(H), supplementary figure 4). Furthermore, all studied frequencies (ranging from 2–200 Hz here) were progressively increased at distances of 8 mm and longer (figures 2 and 4). Notably, our results do not suggest that the underlying biophysical signals are being altered by different re-referencing distances, but rather, the measurement and interpretation is altered.

Previous work on independent component analysis and bipolar reference strategies has suggested that bipolar referencing functions as an approximation of the first spatial derivative [2]. Its effect on the frequency spectrum can therefore be approximated by taking the derivative of the Fourier transform, where the Fourier coefficients complex conjugates are multiplied by the frequency. This results in higher frequencies being amplified and lower frequencies attenuated. Our work critically demonstrates this holds at bipolar referencing distances < 8 mm, but not for larger distances. This may have been hinted at in previous work looking at ECoG signals in the setting of burst suppression, with sequentially larger BPRR distances increasing bipolar re-referenced voltage amplitudes, referred to as ‘burst summation’ at longer distances [40].

We also tested practical uses of these principles: for research, evaluating high gamma range frequencies in the STG during speech perception across BPRR distances, and for clinical contexts, evaluating IED detection between 4–5 mm versus 8–10 mm (similar to recent study on seizure onsets) [12]. A wide range of BPRR distances (4–40 mm in this study) was adequate for the detection of significant behavioral task-dependent increases in high gamma range activity. High gamma activity is a common biomarker in research and emerging clinical brain–machine interfaces, including for speech perception [36] and production [41] as well as motor [42] and even psychiatric applications [21]. Other recent work has explored lower frequency bands and even combinations of frequencies (e.g. phase-amplitude coupling) as a biomarker [43]. Our results show that emerging biomarkers targeted in neuromodulation for neurological dependence including specific ranges of gamma [21] and beta [44] are influenced by the choice of inter-electrode distance during implantation and programming. In other words, the specific distance-dependent influences we observed on BPRR signal analyses warrant special consideration when selecting electrodes for biomarker determination.

We found no consistent significant difference in the quantitative detection of IEDs between the two BPRR distance ranges evaluated (specifically, IED durations and spatial extent across electrodes), when testing various line length durations after Bonferroni correction. Importantly, we assessed this effect with shared electrodes between the two conditions for anatomic within-patient consistency (figure 6(A)). This may be due to the lack of sampling at key tissue margins. For example, the spatial extent of seizure onset zones is significantly increased and closer to the true size when using higher density arrays [12], and work using microelectrodes in humans has demonstrated sub-millimeter microseizure events [15, 45]. Such pathophysiological details may go unobserved with sparser electrode coverage, and likewise, longer BPRR distances [29, 46].

Alternatively, the lack of consistent, clear differences between actual versus subsampled distances (4–5 vs 8–10 mm; figure 6) in the spatial and temporal extents of IEDs could also relate to the quantitative approach used herein. Namely, line length accentuates epileptiform features useful for detection purposes [33, 47], but acts predominantly as a high-pass filter, which may have thus preserved the spatial and temporal profiles of locally generated activity in both conditions. Overall, our results herein suggest that current manufactured inter-electrode distances may be relatively sufficient for clinical IED detection purposes, yet further work on optimizing electrode density and BPRR distances for IED detection is warranted.

Our findings were consistent across grid, strip, and depth components. This suggests that the non-linear spatial influences we observed with BPRR distance are a bio-signal property in physical space and not an artifact of the recording method or neuroanatomical regions sampled. Indeed, anatomically, our coverage sampled across a variety of different lobes, structures (gyri, sulci, fissures), and the diverse angles used in 3D space acted to neutralize structure- or region-specific influences. The consistency of distance-frequency maps across patients, despite different coverage, also underscores this point (figure 4(E)). We did not delineate a difference between contacts in white and gray matter, which are known to have differences in signal content [44], and this was a limitation of this study. Nevertheless, the consistency of our results between grids, strips, and depths speaks to the generalizability of our results as a physical property of electric field distributions of brain bio-signals in 3D space.

The nonlinear and contrasting influences of BPRR distance are intriguingly related to a transition point around 8 mm in distance and < 30 Hz in frequency. We speculate that these distance-frequency interactions may relate to spatiotemporal properties of propagating cortical field potentials (traveling waves), specifically spatial wavelength [48, 49]. For example, a bipolar pair with a distance that spans a 90° difference of spatial wave phase gives substantially more voltage difference (and thus calculated power), than a spatial angle 20 or 45° offset from the direction of propagation. Most cortical traveling waves have spatial wavelength between 40–200 mm, aligning with this assumption [48]. Notably, our omnidirectional BPRR strategy helps thoroughly sample the spatial extent, scale, and orientation to profile these periodic neural signals in the 3D space of the brain.

The long spatial wavelengths of low frequency oscillations in the brain [48–50] might thus explain why low frequencies between roughly 2–20 Hz (delta, theta, alpha, beta) increased more slowly as a function of distance (in other words, as the spatial phase offset increased), whereas frequencies between 20–30 Hz increased in power at shorter distances perhaps as a function of shorter spatial wavelengths. Relatedly, gamma and high gamma frequencies >30 Hz have relatively less oscillatory (periodic) properties, and a larger proportion of aperiodic signals (stochastic aspects of high frequency broadband activity). Less prominence of oscillations would convey less relevance of spatial wavelengths [9]. Periodic vs. aperiodic signal properties may thus explain the distance-dependent contrasts between frequencies below and above ∼30 Hz, the latter of which exhibited a plateau effect already at the shortest distances evaluated (figure 4(F)). In fact, the non-linear aspect of distance-related attenuation observed in this work aligns well with Nunez’s theories regarding alpha oscillations in particular—namely, that the structure and connectivity of the cortex provide conditions particularly suited for emergent propagating oscillations in the alpha range [51, 52]. This could help explain why the effect diminishes at lower and higher frequencies which may have lesser and lesser inherent propagating properties. Indeed, these properties could produce the non-linear crescent-shaped pattern that is seen most clearly in the global comprehensive plot in figure 4(G) for grids, which had by far the most electrode pairs for sampling.

While we studied the power (amplitude dependent) of signals in this investigation, we did not specifically explore the impact of BPRR on the phase of the resultant signals, which would be important for phase-dependent applications such as phase-amplitude coupling. We would speculate that the power/amplitude aspects of cross-frequency coupling would be affected similar to how power was affected by BPRR in our study—for example, if CFC was found on referential signals between theta (phase) and high gamma range (amplitude), and BPRR was applied to high gamma only (amplitude signal) it may be augmented by most BPRR distances relative to using referential signals for high gamma range frequencies. Again however, this may or may not be the case if the signal for phase also undergoes BPRR, since this drastically affects phase measurements. The impact of these re-referencing distances on signal phase would be a point for future investigation. As re-referencing does not change the signal itself, but rather its measurement, it would be important for investigators of cross-frequency coupling to explicitly describe not only their referencing strategy, but also distances for BPRR (as evidenced by the current study), to allow for comparisons between studies.

We focused on interictal and baseline activity windows for clinical applicability. Seizure (ictal) data would be highly relevant as well to understand how distance affects the detection of seizure onsets, yet this is much more limited per patient and definitive conclusions would be underpowered. Nevertheless, we speculate that our IED results (largely containing embedded spike waveforms) would hold for seizures that have a spiking onset. Other important seizure onset patterns, such as low-voltage fast activity, may be similarly influenced by distance; this pattern involves higher frequencies that tended to be augmented regardless of bipolar reference distance (refer to figures 2 and 4) [6]. Regardless, further direct study of influences of the distance of BPRR on seizure detection is warranted to corroborate this speculation.

Our study was further limited in that we did not record from all possible anatomical regions in both hemispheres. However, we used massive numbers of electrodes and bipolar pairs across patients, and our results were generally similar across patients with different coverage (figure 4). Thus, we anticipate similar physical properties would be maintained across anatomical regions not sampled in our cohort. Additionally, this study was performed on digitized signals—it is unclear whether the effects of distance on digital re-referencing would be analogous to bipolar referencing prior to digitization, or other local re-referencing schemes (e.g. laplacian [4]), opening doors for future work. Future work in this space could also take the anatomical features (e.g. specific region, structure) as well as the spatial relation (angle) of the bipolar pair into account.

## 5. Conclusion

This study provides the first systematic quantification of BPRR distance-frequency relations for biomarker power, behavioral neurophysiology, and clinically relevant IED detection. The consistent complex nonlinear relations we uncovered were nevertheless consistent, for which we provide a detailed ‘map’ charting these specific distance-frequency-power relations (figure 4(f)). This work helps explain the physical origins of bipolar signal transformations, and it provides a blueprint for optimizing the SNR of spectral intracranial biomarker signals.

## Data Availability

The code for this project is available at: https://github.com/Kleen-Lab/Bipolar_Expedition. The repository containing the data that support the findings of this study can be accessed at: https://doi.org/10.17605/OSF.IO/CN8W4. Supplementary data file available at https://doi.org/10.1088/1741-2552/ae1b3b/data1.
